# Clinical evaluation of targeted arterial perfusion of verapamil and chemotherapeutic drugs in interventional therapy of advanced lung cancer

**DOI:** 10.1007/s00280-013-2271-1

**Published:** 2013-08-22

**Authors:** Jin Huang, Tengyue Zhang, Kelong Ma, Pingsheng Fan, Yabei Liu, Chengtao Weng, Gaofei Fan, Qiaohong Duan, Xianhai Zhu

**Affiliations:** 1 Department of Medical Oncology, Anhui Provincial Cancer Hospital, Anhui Medical University, 107 Huanhu Road East, Hefei, 230031 People’s Republic of China; 2 School of Life Science, Anhui Medical University, Hefei, 230032 People’s Republic of China; 3 School of Integrated Traditional Chinese and Western Medicine, Anhui University of Chinese Medicine, Hefei, 230038 People’s Republic of China

**Keywords:** Verapamil, Advanced lung cancer, Arterial perfusion, Multidrug resistance

## Abstract

**Purpose:**

To assess the clinical efficacy of targeted arterial perfusion of verapamil and chemotherapeutic agents in the interventional therapy of lung cancer.

**Methods:**

Forty patients with advanced lung cancer underwent treatment with targeted arterial perfusion of verapamil and chemotherapeutic agents using Seldinger technique. Interventional therapy was performed once a month, and each subject received interventional treatment for 2 or more cycles. The therapeutic efficacy was evaluated 2 months post-treatment.

**Results:**

Out of 40 patients with advanced lung cancer, 5 cases achieved complete remission (CR) and 29 cases achieved partial remission (PR), with a total effectiveness (CR + PR) rate of 85 %. Besides, 32 cases achieved significantly alleviated clinical symptoms, and 29 cases had decreased clinical tumor stage. All subjects had stable karnofsky performance status score and body weight. Among the 40 patients, 13 cases had leucopenia, 10 cases had gastrointestinal reactions, 3 cases presented with elevated alanine aminotransferase/aspartate aminotransferase ratio, and 3 cases had fever. However, all these side effects relieved quickly. No elevation of BUN/Cr ratio and allergic reactions was observed. No significant changes in cardiac function and electrocardiogram were noticed after the treatment.

**Conclusions:**

Targeted arterial perfusion of verapamil and chemotherapeutic drugs can improve the clinical symptoms of patients with advanced lung cancer and increase the efficacy of chemotherapeutic agents, thereby providing an opportunity for radiotherapy or surgical treatment for advanced lung cancer.

## Introduction

Lung cancer is one of the most common cancers worldwide, accounting for 1.2 million new cases annually, and the malignancy ranks first in cancer deaths for both men and women [1]. Lung cancer has an occult onset and progresses rapidly. Most of these cases progress to middle or advanced stages at definite diagnosis, thereby losing the opportunity of surgical treatment [2, 3]. As a consequence, adjuvant chemotherapy, notably bronchial arterial infusion (BAI), becomes a major approach for clinical treatment for lung cancer. BAI can directly infuse high-concentration chemotherapeutic agents into tumor tissues, thereby reducing tumor size, improving clinical symptoms, and decreasing the incidence of adverse effects [4]. However, the natural or emergence of insensitivity to chemotherapeutic drugs in lung cancer during treatment, namely chemotherapy resistance, leads to substantially increased failure rates in the treatment for lung cancer, with a total efficacy rate of 40–60 % [5–7]. Use of effective treatment measures to reduce drug resistance in lung cancer and increase the efficacy of chemotherapy is of great significance in improved quality of life and prolonged survival in patients with lung cancer.

Verapamil is a calcium channel blocker, which is mainly indicated for the treatment of heart disease. It has been shown that verapamil significantly reverses the multidrug resistance (MDR) in tumor cells [8], and the concentration of verapamil that can effectively reverse MDR in tumors in vitro is 6.0–10.0 μmol/L. Unfortunately, such a concentration range exceeds the safe concentration (1.0–2.0 μmol/L) of verapamil in the peripheral venous blood [9], however, intravenous administration of verapamil alone cannot reach the concentration large enough to reverse drug resistance. In order to reach the concentration capable of reversing MDR in tumors and avoid cardiac toxicity, targeted arterial perfusion of verapamil and chemotherapeutic agents was performed for tumor chemotherapy. It has been shown in animal experiments that the tissue concentration of verapamil infused via the targeted artery is 3–10 times higher than the blood concentration [10], and verapamil within a safe venous concentration range can reach the concentration that is capable of reversing drug resistance in tumors. Our previous studies showed that targeted arterial infusion of verapamil increased the total effectiveness rate to 71.4 % and 1-year cumulative survival rate to 81.8 % in patients with liver cancer; yet, no cases developed cardiovascular toxicity [11, 12]. It is therefore considered that intra-arterial verapamil infusion can not only avoid the emergence of disorders of the cardiovascular system caused by drug treatment, but also significantly enhance the therapeutic efficacy of chemotherapeutic agents in patients with liver cancer.

In order to further investigate the improvement of intra-arterial verapamil infusion in tumor chemotherapy, and search for effective chemotherapy of advanced lung cancer, interventional therapy with verapamil through the targeted arterial infusion was performed for treatment of advanced lung cancer, and the clinical efficacy of such a treatment regimen in advanced lung cancer was evaluated.

## Subjects and methods

### Subjects

Forty patients with advanced lung cancer who were admitted to our department during the period from March 2010 to March 2013 were enrolled in this study. The subjects included 33 males and 7 females and were aged 32–74 years, with a median age of 61 years. Eleven cases with squamous cell carcinoma of the lung, 10 cases with lung adenocarcinoma, 6 cases moderately differentiated squamous cell carcinoma, 5 cases with clinical diagnosis of lung cancer, 5 cases with mediastinal lymph node metastases from lung cancer, and 3 cases with lung cancer complicated by liver metastasis (Table 1). The participants involved advanced lung cancer patients with failure in standard systemic venous chemotherapy, and some patients undergoing interventional therapy in our department for the first time. This study was approved by the Ethics Review Committee of the hospital. Informed consent was obtained from all participants following a detailed description of the purpose and potential benefits of the study.

**Table 1:** Assessment of therapeutic efficacy in 40 cases

Diagnosis	Therapeutic efficacy					
	Cases					Efficacy
	Total	(CR)	(PR)	(SD)	(PD)	(CR + PR) %
Squamous cell carcinoma	11	1	7	3	0	72.70
Lung adenocarcinoma	10	2	7	1	0	90.00
Moderately differentiated squamous cell carcinoma	6	1	5	0	0	100.00
Clinical diagnosis of lung cancer	5	0	4	0	1	90.00
Mediastinal lymph node metastases from lung cancer	5	1	3	1	0	90.00
Lung cancer complicated by liver metastasis	3	0	3	0	0	100.00
Total	40	3	31	5	1	85.00

The inclusion criteria involved (1) karnofsky performance status (KPS) score ≥70; (2) age range from 18 to 80 years; (3) life expectancy >3 months; (4) heart rate >60 beats/min; (5) patient compliance with the proposed regimen, with informed written consent obtained; and (6) patients with confirmed diagnoses of lung cancer using computed tomography (CT), bronchoscopy, and pathological examination, who had no contraindication to the use of verapamil, who had tumor lesions that could be used for evaluation of therapeutic efficacy, and in whom catheters could be inserted into the feeding arteries of the malignant tumor. Subjects who met the following criteria were excluded from this study: (1) patients with indications for surgery; (2) pregnant or lactating women; (3) patients with mental disorders or oligophrenia; (4) patients with acute infections or symptoms of the central nervous system; (5) patients with a history of allergy; (6) patients with white blood cell (WBC) count <4.0 × 10^9^/L; and (7) patients with blood coagulation disorders. In addition, patients in whom the treatment regimen was not strictly followed, those presenting with serious adverse effects who could not tolerate the study, or patients with incomplete information who presented with difficulty in the assessment of side effects and therapeutic efficacy were withdrawn from the study.

### Interventional treatment method

Interventional therapy was performed once a month, and each subject received interventional treatment for >2 cycles. Efficacy of the interventional treatment was assessed at 2 months post-therapy. In all patients, femoral artery was punctured using the Seldinger technique, and thoracic aortography was performed via a 5-F pigtail catheter to observe the arterial blood supply in the lesion region. Then, a 5-F cobra catheter was implanted into the bronchial arteries that supplied blood for tumors (some were branches of the intercostal artery or branches of the internal thoracic artery), and perfusion therapy was performed [13].

The chemotherapy regimens were developed according to the guidelines for diagnosis and treatment for malignant tumors proposed in 2009 annual meeting of the Chinese Society of Clinical Oncology of Chinese Anti-cancer Association, including the paclitaxel + cisplatin regimen, the docetaxel + cisplatin regimen, the cisplatin and + 5-fluorouracil (5-FU) regimen, and the paclitaxel + gemcitabine + 5-FU regimen. The dose of chemotherapeutic agents was estimated based on the patients’ body surface area and the KPS score.

The perfusion procedures used were as follows: verapamil (15 mg), chemotherapeutic agents, and verapamil (10 mg) again. During the perfusion, the catheters were intermittently flushed with heparinized saline for 3–5 times. Cefoperazone/sulbactam (4.0 g) was infused before and after perfusion of chemotherapeutic agents, and ondansetron (8–16 mg) and dexamethasone (5–10 mg) were intravenously injected simultaneously. Drug doses were identified according to tumor size, patients’ general conditions, and the heart, liver, and kidney functions. Routine symptomatic treatment was performed after surgery, including anti-inflammation, hydration, asthma-relieving treatment, and antiemetic therapy.

### Clinical observation parameters

Routine blood tests, liver and kidney function tests, and electrocardiography (ECG) were performed before treatment and 30 days after treatment, and CT scan was performed before treatment and 30 and 60 days after treatment. Adverse drug reactions, changes in symptoms and signs, changes in KPS score, and changes in body weight were observed and recorded. The clinical benefit caused by 2 cycles of interventional therapy was identified, and the presence of new lesions, changes in primary lesions, changes in KPS score, and body weight changes were observed. Changes in heart rate and blood pressure were monitored before, during, and 5 min after perfusion of verapamil.

### Evaluation of changes in primary lesions

Re-examination with CT scan was performed 3–4 weeks after each treatment. The efficacy of antitumor drugs against solid tumors was assessed according to the response evaluation criteria in solid tumors proposed by the European Organization for Research and Treatment of Cancer, the US National Cancer Institute (NCI) of the National Institutes of Health, and the Canadian Cancer Society Research Institute (CCSRI) [14]. Complete remission (CR): disappearance of all target lesions; partial remission (PR): at least a 30 % decrease in the sum of the longest diameter of target lesion; progressive disease (PD): at least a 20 % increase in the sum of the longest diameter of target lesions, taking as reference the smallest sum longest diameter recorded since the treatment started or the appearance of ≥1 new lesions; stable disease (SD): neither sufficient shrinkage to qualify for PR nor sufficient increase to qualify for PD.

### Identification of clinical benefit

A KPS score increase of >10 which was kept for 4 weeks or longer was defined as a positive clinical benefit, while a reduction in KPS score was defined as a negative clinical benefit; all other outcomes were defined as stable. A body weight increase of more than 7 % which was maintained for 4 weeks or longer was defined as a positive clinical benefit, while a reduction in body weight was defined as a negative clinical benefit; all other outcomes were defined as stable. To be classified as a clinical responder, a patient had to achieve a positive status for at least one of the measures for KPS score or body weight, without being identified as negative for the other.

### Evaluation of toxicity and cardiac function

Toxicities of the anticancer drugs were assessed according to the NCI Common Terminology Criteria for Adverse Events (0–IV) [15], and the cardiac function was classified using the New York Heart Association Functional Classification Criteria (I–IV) [16].

### Statistical analysis

All statistical analyses were performed using the statistical software Statistical Package for the Social Sciences version 16.0 (SPSS Inc., Chicago, IL, USA). All measurement data were expressed as mean ± SD. Differences of proportions were tested for statistical significance with the chi-square test; *t* test was used to compare groups. A *P* < 0.05 was considered statistically significant.

## Results

### Clinical outcomes

**Fig. 1:**
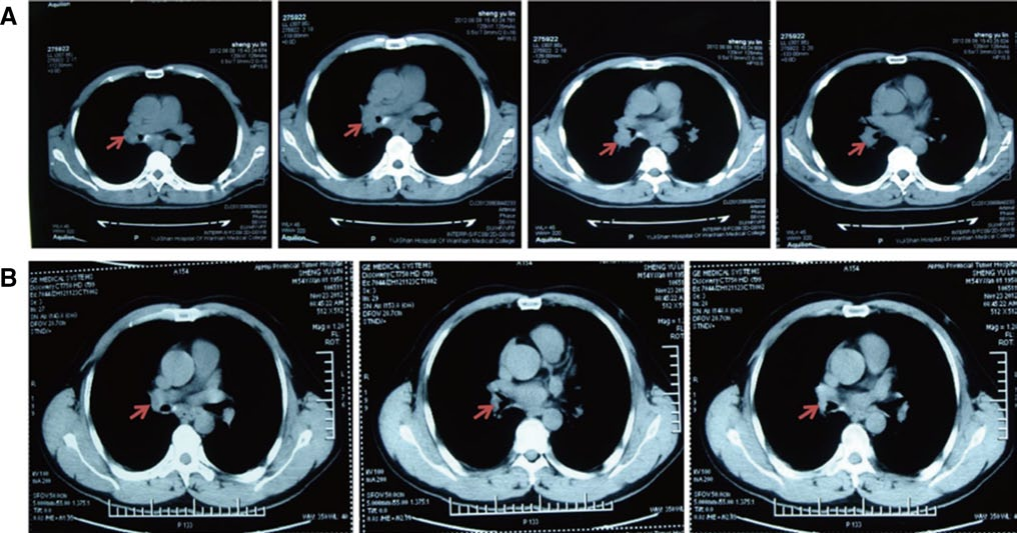
CT scan of the patient with adenocarcinoma of the *right* lung before (**a**) and after (**b**) interventional therapy. The *arrows* showed the position of lesions

**Fig. 2:**
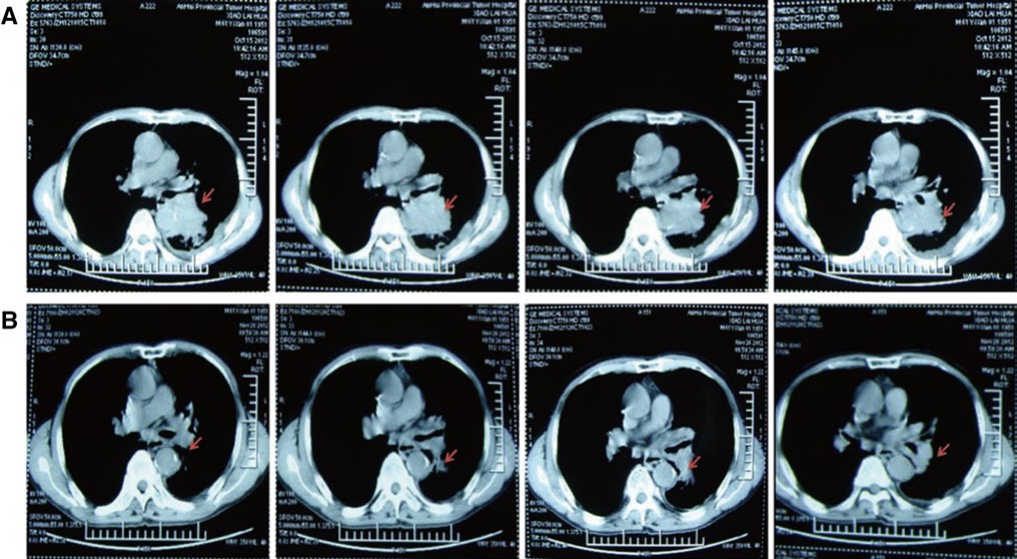
CT scan of the patient with moderately differentiated squamous cell carcinoma of the *left* lung complicated by *right* supraclavicular lymph node metastases before (**a**) and after (**b**) interventional therapy. The *arrows* showed the position of lesions

**Fig. 3:**
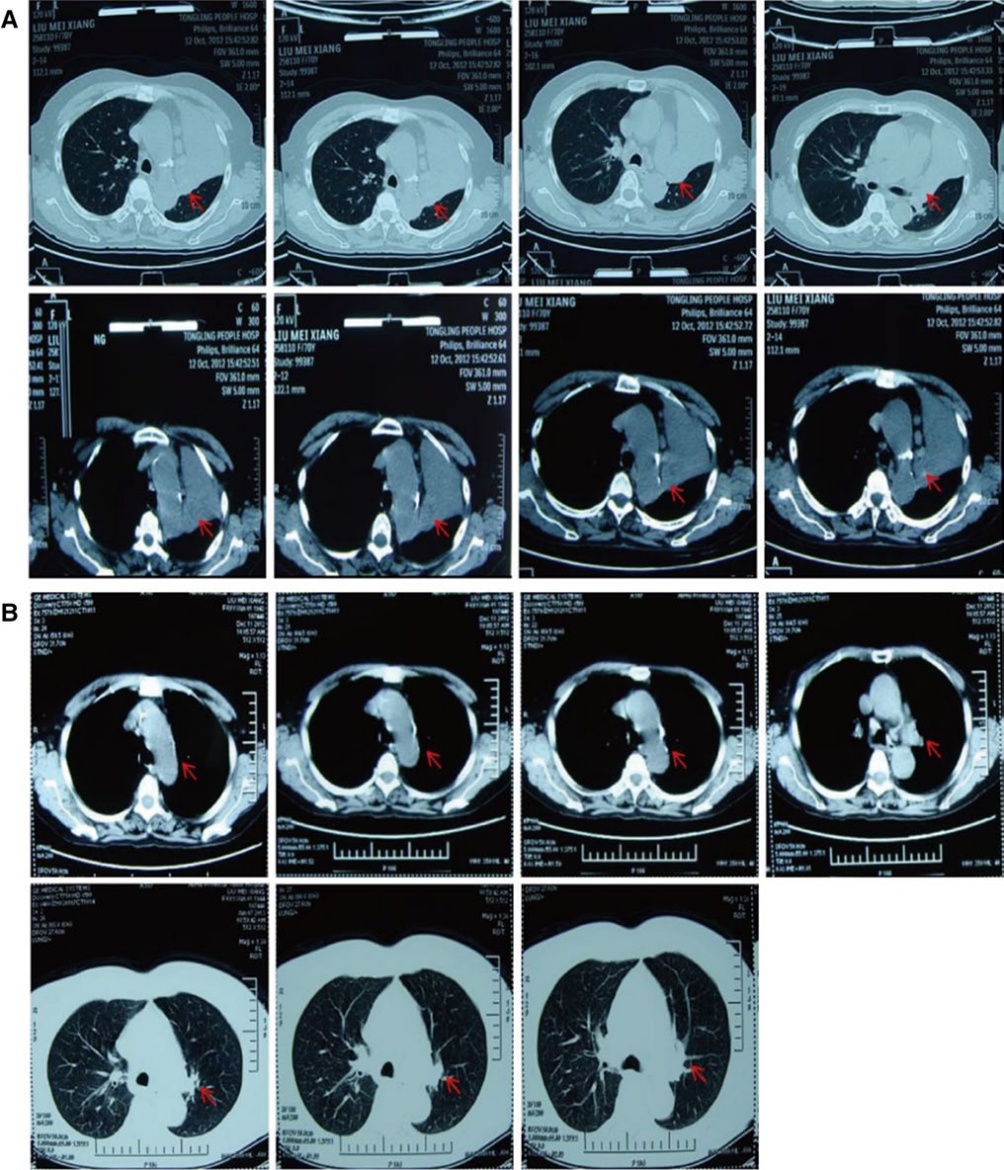
CT scan of the patient with moderately differentiated adenocarcinoma of the *left* lung complicated by atelectasis before (**a**) and after (**b**) interventional therapy. The *arrows* showed the position of lesions

Each of the 40 cases with advanced lung cancer underwent ≥2 cycles of interventional treatment with verapamil and chemotherapeutic agents, while the changes in primary lesions before and after the interventional therapy were evaluated using CT scan 2 months after the treatment. The results showed CR in 5 cases and PR in 29 cases, with a total effectiveness (CR + PR) rate of 85 % (Table 1). In addition, 32 cases achieved significantly alleviated clinical symptoms (e.g., chest pain, cough, and expectoration), and 29 cases had decreased clinical tumor stage, among which 4 cases got chances of surgery and 25 cases of radiation therapy. Notably, following 2 cycles of interventional therapy with verapamil + paclitaxel + gemcitabine + 5-FU in our department, the lesion (at a size of 2.8 cm × 4.1 cm, Fig. 1a) in the lung of a case with adenocarcinoma of the right lung was apparently reduced (at a size of 0.5 cm × 0.8 cm, Fig. 1b), and the symptoms cough and expectoration disappeared, thereby achieving the CR criteria. A patient with moderately differentiated squamous cell carcinoma of the left lung complicated by right supraclavicular lymph node metastases had a PD after 1 cycle of intravenous chemotherapy, and the lesion progressed (at a size of 3.5 cm × 5.2 cm, Fig. 2a) further than before (lesion enlargement and presence of pleural effusion). However, the symptoms cough and expectoration obviously improved, the lesion in the lung reduced by more than 70 % (at a size of 1.1 cm × 1.5 cm, Fig. 2b), and the pleural effusion disappeared after 2 cycles of interventional treatment and 1 cycle of intrapleural chemotherapy. A patient with moderately differentiated adenocarcinoma of the left lung complicated by atelectasis (Fig. 3a) had lung recruitment, got surgical treatment, and achieved pathological alleviation after 2 cycles of interventional therapy with verapamil + docetaxel + cisplatin in our department (Fig. 3b). It is suggested that ≥2 cycles of interventional chemotherapy with verapamil and chemotherapeutic agents can significantly reduce the size of most primary lesions and achieve significantly alleviated clinical symptoms.

### Adverse reactions and cardiac function

Of the 40 patients undergoing ≥2 cycles of interventional chemotherapy with verapamil and chemotherapeutic agents, 13 (32.5 %) cases had leucopenia, 10 (25.0 %) cases had gastrointestinal reactions, 3 (7.5 %) cases presented with elevated alanine aminotransferase/aspartate aminotransferase ratio, and 2 (5.0 %) cases had fever (body temperature, 37.5–39 °C) (Table 2). However, all these side effects subsided quickly. No elevation of BUN/Cr ratio or allergic reactions was observed. None of the subjects had any significant changes in cardiac function before or after the interventional treatment (Table 3). In addition, no obvious changes were detected on ECG (Table 4). The results demonstrated that interventional chemotherapy with verapamil and chemotherapeutic agents altered the clinical symptoms of the advanced lung cancer patients and did not induce severe adverse reactions or cardiac dysfunction.

**Table 2:** Side effects in 40 cases undergoing interventional therapy (*n* = 40)

Side effect	0	I	II	III	IV
Leukopenia	27	10	3	0	0
Reduced hemoglobin level	38	2	0	0	0
Reduced platelet count	39	1	0	0	0
Gastrointestinal reactions	30	7	3	0	0
Elevation of ALT/AST ratio	37	3	0	0	0
Elevation of BUN/Cr ratio	40	0	0	0	0
Fever	38	2	0	0	0
Muscle and joint pain	34	6	0	0	0
Allergy	40	0	0	0	0

**Table 3:** Observation on cardiac function (*n* = 40)

Time	Cardiac functional classification				
	Normal	Grade I	Grade II	Grade III	Grade IV
1 day before treatment	40	0	0	0	0
30 days after treatment	40	0	0	0	0
60 days after treatment	40	0	0	0	0

No significant differences were detected in the cardiac function of the 40 cases before and after interventional therapy

**Table 4:** Observation on electrocardiographic results (*n* = 40)

Time	Observation index			
	PR interval (s)	QT interval (s)	QRS duration (s)	Heart rate (beats/min)
0 min	0.16 ± 0.03	0.36 ± 0.03	0.08 ± 0.01	75 ± 13
5 min	0.16 ± 0.02	0.36 ± 0.03	0.08 ± 0.01	75 ± 12
10 min	0.16 ± 0.03	0.36 ± 0.03	0.08 ± 0.01	75 ± 12
20 min	0.16 ± 0.03	0.36 ± 0.02	0.08 ± 0.01	75 ± 12
50 min	0.16 ± 0.03	0.36 ± 0.03	0.08 ± 0.01	75 ± 12

The time in the table indicated the time after administration of verapamil versus the mean values of the observation indexes at 0 min after administration of verapamil

## Discussion

Multidrug resistance is a major cause of diminished clinical chemotherapy efficacy of cancer therapies, while abnormal cellular efflux pump is a mechanism underlying the emergence of MDR in tumor cells. It is indicated that P-glycoprotein (P-gp) hydrolyzes ATP to generate ADP and release energy and binds to intracellular antitumor drugs with the involvement of calcium ions (Ca^2+^), which pump the antitumor agents out of cells, leading to a reduction in intracellular drug concentration while reducing the efficacy of the antitumor agents, resulting in development of MDR [17, 18]. As a calcium channel antagonist, verapamil is shown to inhibit MDR-1 expression and P-gp synthesis, thereby increasing the concentration of chemotherapeutic agents in tumor cells and overcoming drug resistance in tumor cells [19]. Verapamil at a dose of 6–10 μmol/L is shown to completely inhibit the reversal of MDR by P-gp in malignant tumor cells and enhances the sensitivity of tumor cells to chemotherapeutic agents [9]. However, verapamil at a serum concentration of 1–2 μmol/L may induce adverse effects in the cardiovascular system, including a reduced heart rate [20], which is a major cause that limits the wide application of verapamil as a MDR reversal agent in clinical oncology.

Increased application of interventional radiology in diagnosis and treatment for lung cancer, along with improvement of interventional therapeutic techniques, has made interventional therapy the major treatment for middle-stage and advanced lung cancer. Traditional interventional treatments for lung cancer are performed through administration of chemotherapeutic agents combined with embolization. These therapies suffer from problems due to increases in counts of hypoxic cells in the target radiotherapy region, leading to a reduction in efficacy of radiotherapy. Transcatheter targeted arterial interventional therapy can directly infuse high-concentration drugs into lesion tissues, which produces high concentrations of chemotherapeutic agents in the tumor tissues, and low concentrations in peritumor tissues, as well as other unrelated organs and tissues, resulting in extremely low systemic adverse effects.

Considering these limitations of the venous concentration range of verapamil and its high reversal effect on MDR in tumors, a series of studies to evaluate the efficacy of transcatheter interventional therapy with verapamil and chemotherapeutic drugs have been conducted. It has been found in animal experiments that the tissue concentration of verapamil infused via the targeted artery is 3–10 times greater than the blood concentration, and targeted arterial infusion of verapamil can reverse MDR at a concentration range that does not induce cardiac toxicity [10]. Based on these exciting findings, we then investigated the clinical efficacy of arterial perfusion, transthoracic and intraperitoneal perfusion, and portal venous perfusion of verapamil and chemotherapeutic drugs, and high clinical effect was observed in advanced liver cancer [12], colon cancer [21], gastric cancer, and malignant pleural effusion and ascites [22].

In our study, interventional therapy with verapamil and chemotherapeutic drugs through targeted arterial perfusion was performed for treatment of middle-stage and advanced lung cancer for the first time, and the therapeutic efficacy of the interventional treatment was evaluated through assessment of clinical therapeutic efficacy (clinical symptoms and cancer size), degree of clinical benefit (KPS score and body weight), and adverse reactions (cardiac toxicity, WBC count, and gastrointestinal reactions). Our findings showed significant alleviation of clinical symptoms in most patients after two or more cycles of interventional treatment, with a total effectiveness (CR + PR) rate of 85 % (CR in 5 cases and PR in 29 cases), and significantly reduced cancer sizes in some patients. Following 2 cycles of interventional therapy, the lesion in the lung of a case with adenocarcinoma of the left lung disappeared, and the lesion in the lung of a patient with moderately differentiated squamous cell carcinoma of the left lung complicated by right supraclavicular lymph node metastases reduced by more than 70 % following interventional therapy. Assessment of clinical benefit revealed stable KPS scores and body weight in all 40 subjects, while no abnormal adverse reactions were observed. None of the subjects had any significant changes in cardiac function before or after the interventional treatment, and no obvious changes were seen on ECG. In addition, no obvious adverse reactions of the cardiovascular system were detected. It is concluded that targeted arterial perfusion of verapamil and chemotherapeutic drugs can improve the clinical symptoms of patients with advanced lung cancer, increase the efficacy of chemotherapeutic agents, and reduce the clinical tumor stage, thereby providing an opportunity for radiotherapy or surgical treatment of advanced lung cancer. Further studies to investigate the therapeutic efficacy of interventional treatment with verapamil and chemotherapeutic agents in lung cancer in larger sample sizes seem justified.
